# Report from the World Health Organization's Immunization and Vaccines-related Implementation Research Advisory Committee (IVIR-AC) meeting, virtual gathering, 1–5 September 2025

**DOI:** 10.1016/j.vaccine.2025.127903

**Published:** 2025-12-05

**Authors:** Philipp Lambach, Sheetal Silal, Alyssa N. Sbarra, Mitsuki Koh, Rakesh Aggarwal, Habib Hasan Farooqui, Stefan Flasche, Alexandra B. Hogan, Sun-Young Kim, Kathy Leung, William J. Moss, Allison Portnoy, Meru Sheel, Xuan-Yi Wang

**Affiliations:** a Immunizations, Vaccines and Biologicals, World Health Organization, Geneva, Switzerland; b Modelling and Simulation Hub, Africa, University of Cape Town, Cape Town, South Africa; c Centre for Global Health, Nuffield Department of Medicine, Oxford University, Oxford, United Kingdom; d Johns Hopkins Bloomberg School of Public Health, Baltimore, MD, United States; e Sanjay Gandhi Postgraduate Institute of Medical Sciences, Lucknow, India; f College of Medicine, QU Health, Qatar University, Qatar; g Centre for Global Health, Charite, Berlin, Germany; h School of Population Health, Faculty of Medicine and Health, UNSW, Sydney, Australia; i Seoul National University, Seoul, South Korea; j School of Public Health, The University of Hong Kong, Hong Kong, China; k Department of Global Health, Boston University School of Public Health, Boston, United States; l Center for Health Decision Science, Harvard T.H. Chan School of Public Health, Boston, United States; m School of Public Health, Faculty of Medicine and Health, University of Sydney, Sydney, Australia; n Sydney Infectious Diseases Institute, Faculty of Medicine and Health, University of Sydney, Sydney, Australia; o Shanghai Institute of Infectious Disease and Biosecurity, Fudan University, Shanghai, China

**Keywords:** Vaccination, Immunization, IVIR-AC

## Abstract

Serving as the World Health Organization's (WHO) key advisory body for independently reviewing research that assesses the impact and value of vaccines, particularly using transmission and economic modeling analyses, the Immunization and Vaccines-related Implementation Research Advisory Committee (IVIR-AC) gathered for its second semi-annual meeting of 2025 from 1 to 5 September and complemented by ad hoc sessions on 8–10 July. IVIR-AC provided feedback and recommendations across eleven key sessions. This report summarizes the discussions and outcomes of the meeting. Topics covered included leveraging implementation research in WHO African region, Immunization Agenda 2030 (IA2030) impact modeling, subnational immunization coverage estimates for priority countries, modeling diphtheria-tetanus-and-pertussis burden and vaccination strategies, guidance to translate modeling to support evidence-informed decision-making for immunization, multi-model comparisons for typhoid conjugate vaccine schedules, novel metrics to value combination vaccines**,** stockpiling and vaccination strategies for Ebola response, malaria multi-model comparison of prioritized interventions, additional seasonal doses of malaria vaccine, and 3-dose vs 4-dose malaria vaccine schedule in perennial settings.

## 1 Context, scope and objectives of meeting

Robust evidence is essential to guide policies for vaccination programs. The Immunization and Vaccines-related Implementation Research Advisory Committee (IVIR-AC), an advisory body to the World Health Organization (WHO), evaluates and reviews priority topics (including vaccine effectiveness and impact studies, value assessments and modeling analyses) for the Strategic Advisory Group of Experts on Immunization (SAGE), the Product Development for Vaccines Advisory Committee (PDVAC), and the Immunization, Vaccines, and Biologicals (IVB) Department. IVIR-AC does not have executive or decision-making authority [1]. Through topic-based sessions presented during its semi-annual meetings, IVIR-AC provides expert advice and recommendations to WHO. This report summarizes the discussions and recommendations from IVIR-AC's virtual meeting held on 8–10 July, and 1–5 September 2025 [2].

Eleven meeting sessions were requested by WHO's IVB Department and SAGE to address key topics. The objectives, discussions, and recommendations for each session are summarized in this report.

## 2 Summary of sessions

A full summary for each session is provided in the Supplementary Information.

### 2.1 Session 1: leveraging implementation research in WHO African region

Challenges faced by immunization programs in the WHO African region include lagging immunization coverage in the face of expanding immunization schedules. Immunization delivery strategies rely on “one-size-fits-all” approaches, have limited use of local evidence in strategy implementation, and lack resilience amid declining global health financing.

During the session, IVIR-AC was presented with an overview of challenges faced by immunization programs in the region (e.g., limited sharing of practices across member states) as well as WHO-led immunization implementation research activities in the African region, including SP7 Scoping Project (to inform the “Strategic Priority” within IA2030 with an exploration of vaccine research in the region, including a multi-country landscape analysis and establishing a community of immunization research experts) and the MAINSTREAM initiative (to institutionalize learnings across Gavi country programs). Additionally, a proposed framework for Documenting and Reporting Implementation Strategies of Vaccination Efforts (DRIVE) was presented. IVIR-AC was asked to comment on the DRIVE framework and whether it is suitable for conducting case studies to assess implementation strategies, how the framework could provide highest utility, and how best to promote the framework. IVIR-AC was also asked what role it can play in providing guidance for immunization implementation research in the African region.

Overall, IVIR-AC recognizes the value of immunization implementation research in the African Region to address complex challenges and acknowledges efforts of the SP7 Scoping Project and the MAINSTREAM Initiative in institutionalizing implementation research across member states. IVIR-AC acknowledges the challenges with institutionalizing immunization implementation research in the region and the need for systematic documentation and reporting of innovative implementation strategies. IVIR-AC acknowledges that it can support the African Region in conducting implementation research by periodically reviewing methods and processes, including:

• Prioritization, formulation, and assessment of feasibility of answering implementation research questions
• Assessment of situations in which member states could learn from each other and when local implementation research should be conducted
• Translation of implementation research findings for policy makers
• Identification of necessary skills in implementation research and training opportunities

IVIR-AC agrees that the DRIVE framework provides a comprehensive approach to documenting and reporting implementation strategies but recommends balancing the emphasis on comprehensiveness of reporting with operational feasibility and sustainability. IVIR-AC recommends consulting and socializing the DRIVE framework with key stakeholders (e.g., national public health institutes) to understand its feasibility, utility, and likelihood of uptake, including alignment with other frameworks. IVIR-AC recommends developing operational guidance, including on how reports will be completed, stored, transmitted, collated, synthesized, disseminated, and presented to policymakers. Strategies to operationalize the DRIVE framework could include establishing an electronic data reporting and visualization portal.

### 2.2 Session 2: IA2030 impact modeling

In 2021, WHO IVB produced the first iteration of modeled vaccine impact estimates as part of the Immunization Agenda 2030 (IA2030) Monitoring & Evaluation framework. The IA2030 impact modeling team aims to produce target estimates and establish an updated annual reporting process in advance of the WUENIC release in 2026. New estimates will be influenced by updated methodology for extrapolation to non-VIMC countries and updated input data (VIMC estimates and WUENIC coverage data). IVIR-AC was requested to provide guidance on how to communicate changes in results and feedback on the implementation of their previous recommendations [3] on geographical imputation of VIMC-based estimates, incorporating influenza impact estimates into the analytical framework, and switching from the calendar year-based approach to year-of-vaccination based approach for polio.

During the session, the team presented updated project methods based on previous feedback from IVIR-AC. Changes included implementing year-of-vaccination impact calculations for select pathogens, reducing the number of potential statistical models, including sociodemographic index as a predictor in models, and exploring the importance of region and income level in models. Ultimately, the team found that including random effects for WHO region and income level improved the model fit. IVIR-AC emphasizes the need for comprehensive documentation of the strengths and limitations of the current IA2030 impact methodology, including, but not limited to, the year-of-vaccination approach, differences in extrapolation across pathogens, and the variation in estimated impact across project updates. This documentation should capture the evolution of the IA2030 methods and outputs from inception.

The team outlined methodology to estimate results for additional pathogens that are now included in the evaluation framework. Influenza estimates were added for current and no-vaccination scenarios for years 2025 and onward based on work from the London School of Hygiene and Tropical Medicine. As the influenza estimates need to be extrapolated for years 2020 to 2024, IVIR-AC suggests testing output sensitivity to the assumption related to applying the estimated impact factor to the preceding five years. Polio estimates from KidRisk, along with coverage estimates of oral and inactivated polio vaccines, were additionally added through 2023. Finally, additional VIMC pathogens were added including malaria, cholera, typhoid, and COVID-19. IVIR-AC recommends investigating the magnitude of the COVID-19 vaccine impact estimates, particularly with respect to the year-of-vaccination impact method and volume of doses delivered.

Additional project updates included using updated model runs across VIMC pathogens and updated WUENIC coverage estimates. IVIR-AC recommends an iterative approach in communication to global, regional, and national stakeholders. With careful consideration, this communication should outline the multiple analysis stages and updates, convey uncertainty, summarize changes in methodology, and address notable changes in impact estimates.

### 2.3 Session 3: immunization coverage estimates – subnational estimates for priority countries

The WHO/UNICEF estimates of national immunization coverage (WUENIC) currently only provide country-level estimates and for one age cohort. There is interest in expanding WUENIC to produce subnational coverage estimates at the first-administrative unit level for large priority countries and to monitor catch-up vaccination (i.e., children vaccinated after the routine target age). IVIR-AC was asked to provide feedback on the proposed next steps for the estimation of subnational coverage, potential risks, and criteria to determine feasibility of subnational estimation. Additionally, IVIR-AC was asked for feedback on the next steps proposed to monitor vaccination coverage and timeliness in cohorts of children.

During the session, the team presented “WUESNIC”, a WUENIC-like exercise to estimate subnational coverage estimates at the first-administrative unit level. A pilot exercise of this approach was conducted for the Democratic Republic of Congo at the first-administrative unit level. Key data included a vaccination coverage survey, 2023 Demographic and Health Survey (DHS) Key Indicator report, and official country estimates. The pilot exercise highlighted data quality concerns, including stock-outs, high dropout rates, underestimated denominators, variability in survey details available, and vaccination card availability. IVIR-AC recommends defining the methods necessary to estimate subnational coverage. Prior to estimation for high-impact countries, these methods should be piloted in a “pathfinder” country with high-quality data to inform the minimally sufficient data requirements for subnational coverage estimation; this pathfinder country may either be one of the high-impact countries or another country. As a flexible approach may be needed to accommodate different settings, IVIR-AC recommends that evaluative criteria for proceeding with subnational coverage estimation include data quality (i.e., Grade of Confidence) and data availability requirements that are suitable across a range of high-impact countries. IVIR-AC recommends careful consideration of whether limited standardization in data triangulation and uncertainty estimation can overcome differing source data availability and quality across individual countries, which may include a revision of the Grade of Confidence criteria. IVIR-AC recommends assessing the feasibility of replicating subnational coverage estimates over time (e.g., whether this exercise could be performed annually) to inform whether continuation of first-administrative unit level estimation efforts is sustainable.

The team also presented the need for a new way to estimate cohort-based coverage to monitor late or catch-up vaccination. However, the data available for this approach are highly variable by country and region. The project team proposed to revise the electronic joint reporting form (eJRF) for reporting vaccination coverage. Overall, IVIR-AC supports the planned efforts to assess the feasibility of estimating cohort coverage metrics through revisions to the eJRF. To carry out these efforts, IVIR-AC recommends providing and using a standard definition of catch-up and delayed vaccination that is acceptable across regions. Furthermore, these definitions and the data requirements for measuring timeliness and cohort-level coverage should be clearly communicated in the revised eJRF.

### 2.4 Session 4: modeling diphtheria-tetanus-and-pertussis burden and vaccination strategies

To increase the duration of protection, the WHO recommends that three booster doses of diphtheria-tetanus-pertussis-containing vaccine (DTPCV) be offered in early childhood, childhood, and adolescence. Evidence to inform country-level decision-making on DTPCV booster doses is needed, especially considering the availability of support for Gavi-eligible countries. As such, a new model and decision support tool (DTP Boost) has been developed. IVIR-AC was asked for feedback on the scope, methodology, use, and dissemination of the tool, how to overcome challenges in model calibration related to data availability or limitations, whether tool functionality to include seroprevalence data may be useful, and—for diphtheria—measuring increased population protection.

During the session, the project team presented the DTP Boost tool as the first interactive, live-running web-based decision-making tool that integrates health and economic effects of DTPCV booster dose introduction and routine delivery, which was piloted in Uganda. IVIR-AC acknowledges that the methodology and presentation of the DTP Boost tool is logical and well-considered, the interface is well-designed, and the tool is generally intuitive to use for those with modeling expertise.

The tool was designed to be used in collaboration between modelers and country partners, and produces estimates including cases, deaths, cost-effectiveness, and budget impact across scenarios of primary series, booster(s), and maternal vaccine delivery across different schedules, coverage levels, and delivery locations. IVIR-AC agrees that the tool should only be used in collaboration with modelers and technical experts. IVIR-AC recommends that model calibration and validation be performed separately to the online tool, and that the primary objective of the tool should be to facilitate exploration and discussion of vaccination scenarios. IVIR-AC recommends that model calibration and validation be substantially improved, including by:

• expanding model validity checks with additional output metrics (e.g., direct and indirect effects of vaccination) to ensure that the model produces a reasonable fit to the data;
• clearly communicating how the model was calibrated and demonstrating that the model adequately captures local disease epidemiology; and
• featuring uncertainty more prominently, such as by conducting and communicating probabilistic sensitivity analyses (PSA).

For user inputs that are difficult to estimate (e.g., reporting and treatment ratios), IVIR-AC recommends that consideration be given to parameters that could be integrated within the calibration procedure, which could include leveraging serologic data. IVIR-AC notes that capturing the risk of diphtheria outbreaks would likely require a stochastic model, and that transparently communicating assumptions about outbreak risk and vaccine impact in the current framework would be difficult. It may be appropriate to only present population protection and doses required as the outputs for diphtheria.

### 2.5 Session 5: WHO guidance to translate modeling to support evidence-informed decision-making for immunization

In 2023, IVIR-AC recommended development of WHO guidance document to support the translation of vaccine impact modeling into immunization decision-making [4]. In response, an IVIR-AC subgroup has progressed on developing this guidance, including completing a qualitative needs assessment study [5] and initiating a country implementation plan with key partners [6]. The subgroup has also begun planning capacity-building initiatives for national immunization decision makers and seeks the committee's review of the draft guidance document to be published by the end of 2025. The IVIR-AC subgroup requested a review of the draft guidance document by the full committee, to obtain feedback on the content, perspective and tone of the writing, the current format of the document, including plans for an online appendix and what content might be best suited for the online appendix versus main document, and how components of the overall package of resources can best complement each other.

During the session, an overview of the guidance document was presented. The guidance document has been reviewed throughout 2025 by regional immunization technical advisory group (RITAG) members, national immunization technical advisory group (NITAGs) members, global and regional WHO experts, and other stakeholders.

Overall, IVIR-AC commends the team for developing a comprehensive guidance document to support the use of modeling for immunization decision-making. IVIR-AC agrees that the technical content, style, and tone of the guidance document are appropriate for decision makers to use as a resource.

The writing team presented each of the four chapters of the guidance document: (1) “What are models and how can they help us?”; (2) “How can policy makers work together with modelers?”; (3) “How are models made?”; and (4) “How should modeled results be interpreted and evaluated?”. IVIR-AC recommends structural and editorial suggestions to improve the flow and usability of the guidance document, including outlining principles and guidelines when models should versus should not be used, providing information on how to initiate a modeling collaboration, and citations to other sections within the document. Given the intended end users of the document are decision makers, IVIR-AC recommends that only a high-level summary of the technical chapter (i.e., “How are models made?”) be included in the main guidance document and a full description be included in the online appendix.

Future plans for the IVIR-AC subgroup include incorporating IVIR-AC recommendations, additional review of the guidance document, document finalization, development of online web appendix material, and identifying possible training opportunities. IVIR-AC recommends early engagement and planning for a launch and dissemination plan to ensure broad awareness and uptake of the guidance document.

### 2.6 Session 6: WHO multi-model comparisons for typhoid conjugate vaccine adequate schedules (MMC-TAS)

Based on emerging evidence of waning effectiveness of the typhoid-conjugate vaccine (TCV) four to five years after the primary dose, SAGE is currently revising the policy guidance of TCV use for countries that have introduced or plan to introduce TCV in their routine program. To assess the epidemiological, economic, and equity impact of different TCV schedules in different country settings, a multi-model comparison analysis has been undertaken to develop robust model-based evidence on the optimal TCV schedule as an input to SAGE deliberations scheduled for March 2026. IVIR-AC was asked to provide feedback on the implementation of their previous recommendations [3], harmonization of data (including the interpretation of the potential outliers from the different models), and how to interpret and translate the multi-model results including uncertainty for deliberations of the SAGE WG to support their policy recommendations.

During the session, results of the multi-model comparison analysis were presented. The comparison includes four models used to project various TCV delivery strategies, under different coverage assumptions: no vaccination, single dose routine vaccination, and single dose routine vaccination plus booster dose(s). For each delivery strategy, models were run across two waning scenarios (i.e., “fast” and “slow”), three burden archetypes (i.e., medium, high, and very high incidence), and two settings (i.e., Africa with higher case fatality rates (CFR) and treatment costs, and Asia with lower CFR and treatment costs). IVIR-AC acknowledges the updates by the modeling groups to respond to the previous recommendations [3], including PSA and investigating individual-level vaccine responses. IVIR-AC recommends exploring stratification of burden scenarios by mortality.

Results suggested that over a 10-year time horizon, per 100,000 people in a high incidence setting with approximately 200 cases per 100,000 people per year, TCV introduction can avert 250–1500 cases (5–45 cases per 1000 doses), 15–300 hospitalizations, 0.5–32 deaths, and 25–2000 DALYs. TCV introduction is cost-effective at a willingness-to-pay threshold of approximately $2500 (i.e., the average gross domestic product (GDP) per capita for a lower-middle-income country) when incidence is greater than 10–60 cases/100,000 person-years in Africa and greater than 75–200 cases per 100,000 person-years in Asia; adding a booster dose at 5 years old is cost-effective when typhoid incidence is greater than 100–250 cases per 100,000 person-years in Africa and greater than 500–1000 cases per 100,000 person-years in Asia. Given differing optimal strategies predicted across models for the alternative waning and burden scenarios, IVIR-AC recommends that the key drivers and uncertainties generating these differences be transparently communicated. Differences in estimated impact and optimal strategies should be explained in the context of underlying model structure, data used for calibration, incorporation of waning assumptions, and plausibility of incidence estimates in the no-vaccination scenario. In addition to cost-effectiveness acceptability curves, IVIR-AC recommends presenting model output in relation to incidence reductions over time across models, scenarios, and time horizons to improve interpretability. IVIR-AC recommends presenting the current cost-effectiveness acceptability curves in conjunction with the key uncertainty drivers from each model contributing to the observed results (e.g., tornado diagrams).

### 2.7 Session 7: novel metrics to value combination vaccines

Combination vaccines offer promise to reduce logistical burdens for existing vaccines and enhancing the value proposition of new vaccines, particularly those with moderate individual impact. However, there are currently limited research and development (R&D) priorities, regulatory guidelines and no established pathway for recommendations on the development and use of combination vaccines. WHO and PATH are developing a framework to identify, assess the value of, and prioritize novel combination vaccines for endemic pathogens. IVIR-AC was asked to provide feedback on the current and proposed value drivers and metrics to assess the value of novel combination vaccines, and on a checklist of prioritized value drivers and associated metrics to enhance comprehensiveness of combination vaccine evaluations.

The project team presented results of a non-systematic literature review conducted to establish a baseline understanding of how combination vaccines have been evaluated to date. The review identified standard health economic metrics often used (e.g., reduction in cases, cost per DALY averted), and those evaluations only described standard incremental benefits and costs of additional antigens rather than comprehensive value added by the combination. Overall, IVIR-AC agrees that proposing a new set of value drivers and metrics is a necessary and valuable step that will enhance the comprehensive evaluation of combination vaccines.

The team also conducted consultative stakeholder engagement sessions to develop list of benefits, value drivers and potential metrics that can be used to capture the full value of combination vaccines. Key areas highlighted included the need to account for risks (not just benefits), costs, market fragmentation and potential loss of autonomy over immunization schedules. IVIR-AC agrees that considerations be made that acceptance and ongoing use of the metrics will be dependent on data availability, maturity of the vaccine products, and widespread acknowledgement and communication of the value of the metrics. IVIR-AC reiterates [3] the need to examine potential risks and unintended consequences of combination vaccines.

Finally, the project team convened health economic experts to prioritize value drivers and metrics based on four criteria (resonance, magnitude, quantifiability, and fit within existing frameworks). Meeting output included a prioritized “checklist” of consensus benefits, value drivers, and metrics for consideration when evaluating combination vaccines. Given the increased complexity of and challenges with evaluating proposed metrics, IVIR-AC recommends determining a prioritized short-list of value drivers and metrics. IVIR-AC proposes that the prioritization framework consider adding dimensions of predictability and generalizability, in order to capture complexity beyond post hoc quantifiability in decision-making. IVIR-AC encourages the use of prioritized value drivers and associated metrics, including applying and using the framework, launching a call for case studies employing these metrics, and reaching out to interest groups of analysts. IVIR-AC recommends continued engagement with RITAGs, NITAGs, and EPI managers to examine data availability to inform framework implementation and additionally communicate findings.

### 2.8 Session 8: stockpiling and vaccination strategies for Ebola response: insights from integrated epidemiological and epidemic modeling

In 2021, a global stockpile of Ebola vaccine was established to ensure equitable, timely, and targeted access to vaccine doses for future Ebola outbreaks. To inform International Coordinating Group (ICG) and Gavi investment decisions, it is imperative to estimate the benefits and costs of alternative vaccination strategies, the optimal size of the stockpile to respond to the most likely outbreak scenarios, and the vaccine price for the intervention to be considered cost-effective in different target populations. To do this, it was necessary to update an existing model of Ebola transmission, and consider scenarios designed to explore a range of plausible parameters, control measures, and vaccination strategies. IVIR-AC was asked to provide feedback on the mathematical and economic model structure, epidemiological and economic parameter ranges, how to assess whether a given stockpile strategy is successful, and effective strategies to communicate technical details of the approach and results.

The modeling team presented an individual-based transmission model that was used to stochastically simulate health outcomes while applying different interventions including healthcare worker vaccination (preventative or reactive) and various case finding-related actions (i.e., contact tracing, ring or geographical vaccination, treatment unit admissions, safe and dignified burials). IVIR-AC agrees that the mathematical and economic model structures presented, which allow exploration of Ebola transmission and intervention scenarios, are appropriate. IVIR-AC recommends that consideration be given to prediction of outbreaks across varying geographic areas (e.g., rural versus urban), demography (e.g., high versus low population density), and temporal windows and to related key parameter assumptions (e.g., transmission, urbanization, contact tracing, vaccine uptake and effectiveness), since the outbreak size can vary widely with variations in these parameters. IVIR-AC notes that defining the success of a stockpile will vary based on the number of simultaneous outbreaks, maintenance of a stockpile, and rate of vaccine delivery and stockpile replenishment.

Results suggested that reactive ring vaccination tends to be cost-saving if at least one large outbreak occurs during a 5-year period and that ring vaccination uses fewer vaccine doses in scenarios with realistic and best-case outbreak responses. The cost-effectiveness of healthcare worker vaccination mainly depended on the frequency of outbreak emergence. IVIR-AC suggests presenting both the percentage of scenarios for which the stockpile size would be sufficient and the assumptions regarding the rate of doses delivered per day. IVIR-AC recommends presenting the impact of the most influential parameters (e.g., changing epidemiology, response, and cost) on the model results. Models could include prioritization of at-risk groups as part of potential vaccine strategies. IVIR-AC recommends distinguishing between the intervention parameters and epidemiological features of transmission as key drivers of impact uncertainty.

### 2.9 Session 9: malaria multi-model comparison of prioritized interventions (M3CPI)

To inform WHO guidance on prioritization of malaria interventions under resource constraints based on SAGE and Malaria Policy Advisory Group (MPAG) advice, WHO is proposing a multi-model analysis to analyze the relative impact and cost of the combinations of various malaria interventions, with assumptions about performance and coverage, in different scenarios in Sub-Saharan Africa. A second technical consultation with involved modeling groups occurred in June 2025. Final project results are expected to be presented to SAGE and MPAG in November 2025. IVIR-AC was requested to provide feedback on the methods for modeling malaria intervention prioritization (including approach for model comparison), recommendations on how to effectively present and communicate results to support decision-making by SAGE and MPAG, specific guidance of the translation of modeling results into WHO guidance on prioritization of malaria interventions document with explicit consideration of the target audience, and recommendations on priority areas for expansion in future phases of the project.

A member of the modeling team presented methods for the multi-model comparison. Across two models, interventions were simulated across first-administrative unit settings using setting-specific input characteristics including infection history, intervention efficacy, and geography. IVIR-AC notes that the two models are well-established, use different sources for parameterization, and, therefore, span a helpful part of the structural and parametric uncertainty space.

For each intervention, 24 future scenarios with scaling up or down of interventions were compared with business as usual. Simulation output over short- and long-term time horizons will include incidence, cases, deaths, and disability-adjusted life years (DALYs) averted combined with estimated costs of interventions (provided by WHO) for cost-effectiveness analysis. IVIR-AC recommends continued adherence to the WHO multi-model comparison guidelines [7], including the exploration of qualitative differences in model predictions and the uncertainty of the combined effect of the interventions.

Proposed visuals across scenarios included Sankey diagrams ranked by probability of cost-effectiveness. IVIR-AC agrees that Sankey diagrams have potential value for communication of results and suggests inclusion of cost differentials of the intervention strategies. Translation of results should be aligned with WHO guidelines for subnational tailoring of malaria interventions [8].

Results to be presented and finalized in November 2025 during 3rd Technical Consultation. In future phases of the project, IVIR-AC suggests priority areas for expansion include to:

o evaluate optimal intervention strategies under a fixed budget as an alternative to the currently pursued identification of the next most cost-effective incremental addition to the set of interventions
o evaluate the benefit of geographic resource re-allocation within country for optimization of impact
o investigate minimum cost strategies for achieving elimination targets

### 2.10 Session 10: additional seasonal doses of malaria vaccine (7 doses vs. 5 doses)

Building on a previous analysis of RTS,S seasonal vaccination, modeling of additional seasonal doses of malaria vaccine was conducted to estimate the public health impact and cost-effectiveness of a 7-dose schedule vs. a 5-dose schedule when administered in a seasonal or hybrid approach. In addition, models were used to estimate the public health impact and cost-effectiveness of different dose allocation strategies, vaccinating fewer children with a 6- or 7-dose schedule or vaccinating more children with a 5-dose schedule. IVIR-AC was asked to provide feedback on additional analyses or model changes to improve the model representation of vaccine efficacy or effectiveness and on the methodological approach for analyzing the comparative public health impact and cost-effectiveness.

The project team presented a model that included extended Phase 3b trial data and was parameterized over a range of transmission levels and a 15-year time horizon. Across a range of seasonal and transmission settings, preliminary model results suggested that both 5-dose and 7-dose RTS,S schedules show positive public health impact and cost-effectiveness relative to no vaccination. Estimated incremental cost-effectiveness ratios (ICERs) remain similar between 5- and 7-dose schedules, as the additional costs of extra doses offset cost savings due to cases averted and were compared across 0 % and 75 % coverages of seasonal malaria chemoprevention (SMC). Given the examination of the incidence rate ratio of RTS,S to SMC in the validation exercise, IVIR-AC notes that it may be relevant to consider scenarios of SMC coverage in addition to 0 % and 75 %.

Preliminary results suggest that allocating doses to reach more children with a 5-dose schedule generally improves health outcomes in children under five but does not provide significant additional benefits when measured in children up to age 10. IVIR-AC recommends additional scenarios for the allocation analysis to include archetypical settings of varied transmission and coverage limitations.

Given a sizeable task of addressing many areas of uncertainty (e.g., model selection, dose schedule, seasonality, transmission intensity, delivery strategy, vaccination coverage), IVIR-AC notes the importance of clearly defining all scenarios and comparisons throughout the reported results, particularly when asserting optimal decision-making. As the optimal choice under consideration is between 5-dose and 7-dose strategies, IVIR-AC urges the research team to present only an incremental cost-effectiveness analysis where these strategies are directly compared against each other. Furthermore, cost-effectiveness thresholds must be included in order to determine the optimal strategy. When estimating the health impact of the 5-dose and 7-dose strategies compared to a no-vaccination scenario, IVIR-AC recommends that the impact on immunity through differences in natural infection rates be explored with a sensitivity analysis.

### 2.11 Session 11: 3 doses vs. 4 doses of malaria vaccine in perennial settings

Modeling has been conducted to compare 3-dose versus 4-dose schedules of malaria vaccine to estimate the potential public health impact and cost-effectiveness across these schedules and to estimate the potential public health impact of different vaccine allocation strategies, such as vaccinating fewer children with 4 doses or more children with 3 doses. Updated model calibration and validation was conducted with additional data made available since previous modeling analyses, including 46-months of follow-up from the malaria vaccine implementation program (MVIP), a case control study embedded within the MVIP, and 2-years of follow-up from the R21 Phase 3 trial. IVIR-AC was asked to provide feedback on the recommended analyses or model changes that should be explored to improve the model representation of vaccine efficacy or effectiveness, methods for analyzing the comparative public health impact and cost-effectiveness, and the model validation process. Additionally, IVIR-AC was asked to review interim results related to calibration of the models to the severe malaria outcomes from the MVIP case-control study. Interim analyses from two models were presented.

IVIR-AC recommends that methods align on vaccination coverage parameters and reporting results for the same age groups, time horizons, and denominators. IVIR-AC suggests that models be fitted separately to data for sites with age-based vaccination (in perennial vs seasonal settings) and seasonal vaccination in seasonal settings. For the Malaria Vaccine Pilot Evaluation (MVPE) analysis, IVIR-AC advises conducting additional analyses to provide better fit to the R21 trial data, including obtaining insights into study data and exploring biological mechanisms that are unaccounted for in the model structure. As the optimal choice under consideration is between 3-dose and 4-dose strategies, IVIR-AC urges the research team to present only an incremental cost-effectiveness analysis where these strategies are directly compared against each other. Discounting must also be included.

Preliminary modeling analysis suggests that between the different allocation strategies, there is no superior strategy for RTS,S. IVIR-AC recommends that the allocation analysis consider whether the different dose allocation strategies are feasible to be delivered to the target group by examining the population size and coverage achieved for other vaccines. For this model, preliminary analysis using an equivalence framework suggested that an allocation strategy was neither inferior nor superior for both vaccines and that any cost savings expected were marginal. IVIR-AC recommends conducting a sensitivity analysis to assess the impact of the estimated faster decay rate in the RTS,S case-control study on the equivalence analysis.

## 3 Recurring themes

• Improvements in surveillance and data quality are essential to strengthening the validity of disease models. These data enable robust estimation of coverage gaps, improve model calibration, and strengthen scenario projections, thereby supporting immunization policy and implementation. This was highlighted in sessions including implementation research in the African region, subnational coverage estimation, DTP Boost tool, and Ebola vaccination strategies.
• Collaborative engagement with and across teams of in-country partners is critical to understand barriers to uptake of modeling and implementation research to improve immunization systems. This was highlighted across sessions including implementation research in the African region, subnational coverage estimation, DTP Boost tool, WHO guidance for translating modeling for decision-making, and metrics to evaluate combination vaccines.
• Comprehensive documentation, including on scenarios, assumptions, parameters, and methods updates, is essential for model evaluation and communication to both technical and non-technical audiences.

IVIR-AC will next convene for its bi-annual meeting from 23 to 27 February 2026.

## CRediT authorship contribution statement

**Philipp Lambach:** Conceptualization, Project administration, Supervision, Writing – review & editing. **Sheetal Silal:** Conceptualization, Investigation, Writing – review & editing. **Alyssa N. Sbarra:** Conceptualization, Writing – original draft. **Mitsuki Koh:** Project administration, Writing – review & editing. **Rakesh Aggarwal:** Investigation, Writing – review & editing. **Habib Hasan Farooqui:** Investigation, Writing – review & editing. **Stefan Flasche:** Investigation, Writing – review & editing. **Alexandra B. Hogan:** Investigation, Writing – review & editing. **Sun-Young Kim:** Investigation, Writing – review & editing. **Kathy Leung:** Investigation, Writing – review & editing. **William J. Moss:** Investigation, Writing – review & editing. **Allison Portnoy:** Investigation, Writing – review & editing. **Meru Sheel:** Investigation, Writing – review & editing. **Xuan-Yi Wang:** Investigation, Writing – review & editing.

## Declaration of competing interest

The authors declare the following financial interests/personal relationships which may be considered as potential competing interests: P. L. is supported by the Gates Foundation. S. S. was supported by the World Health Organization for this work. A. N. S. was financially supported by the World Health Organization for this work, and is additionally supported by the Gates Foundation, Gavi, the Vaccine Alliance, and the National Institutes of Health. M. K. is supported by the Gates Foundation. A. B. H. was supported by the Australian National Health and Medical Research Council for this work, is additionally supported by PATH, the World Health Organization, and Gavi, the Vaccine Alliance, and has received consulting fees from the Australian NSW Ministry of Health, WHO Europe and Asian Development Bank. A. P. is supported by Gavi, the Vaccine Alliance, Imperial College London, the Gates Foundation, and the World Health Organization. A. N. S. and A. B. H. report travel related support from the World Health Organization to attend previous IVIR-AC meetings. All other authors have no declarations.

## Data Availability

No data was used for the research described in the article.

## Appendix A Supplementary data

Supplementary material Full description of sessions. Supplementary material
