# Erratum to “Report from the World Health Organization's Immunization and Vaccines-related Implementation Research Advisory Committee (IVIR-AC) meeting, virtual gathering, 1–5 September 2025” [Vaccine 68 (2025) 127903]

**DOI:** 10.1016/j.vaccine.2025.127972

**Published:** 2026-01-01

**Authors:** Philipp Lambach, Sheetal Silal, Alyssa N. Sbarra, Mitsuki Koh, Rakesh Aggarwal, Habib Hasan Farooqui, Stefan Flasche, Alexandra B. Hogan, Sun-Young Kim, Kathy Leung, William J. Moss, Allison Portnoy, Meru Sheel, Xuan-Yi Wang

**Affiliations:** a Immunizations, Vaccines and Biologicals, World Health Organization, Geneva, Switzerland; b Modelling and Simulation Hub, Africa, University of Cape Town, Cape Town, South Africa; c Centre for Global Health, Nuffield Department of Medicine, Oxford University, Oxford, United Kingdom; d Johns Hopkins Bloomberg School of Public Health, Baltimore, MD, United States; e Sanjay Gandhi Postgraduate Institute of Medical Sciences, Lucknow, India; f College of Medicine, QU Health, Qatar University, Qatar; g Centre for Global Health, Charite, Berlin, Germany; h School of Population Health, Faculty of Medicine and Health, UNSW Sydney, Australia; i Seoul National University, Seoul, South Korea; j School of Public Health, The University of Hong Kong, Hong Kong SAR, China; k Department of Global Health, Boston University School of Public Health, Boston, United States; l Center for Health Decision Science, Harvard T.H. Chan School of Public Health, Boston, United States; m School of Public Health, Faculty of Medicine and Health, University of Sydney, Sydney, Australia; n Sydney Infectious Diseases Institute, Faculty of Medicine and Health, University of Sydney, Sydney, Australia; o Shanghai Institute of Infectious Disease and Biosecurity, Fudan University, Shanghai, China

The publisher sincerely regrets the incorrect publication of the authors' affiliation details. The accurate affiliation information is provided above.

The publisher would like to apologise for any inconvenience caused.

