# Supplementary material for: Report from the World Health Organization's Immunization and Vaccines-related Implementation Research Advisory Committee (IVIR-AC) meeting, virtual gathering, 1–5 September 2025
Source: Vaccine. 2025 Dec 5;68:None. doi: 10.1016/j.vaccine.2025.127903 (PMC12683323; doi:10.1016/j.vaccine.2025.127903)
Supplement: Supplementary material — Full description of sessions. [file mmc1.docx]

# Supplementary Material

## Report from the World Health Organization’s Immunization and Vaccines-related Implementation Research Advisory Committee (IVIR-AC) meeting, virtual gathering, 1 – 5 September 2025

Extended session summaries

*Session 1: Leveraging implementation research in WHO African region*

Colleagues from the WHO African region highlighted the importance of implementation research to inform effective and equitable implementation strategies in the region and understand its role in the broader immunization research ecosystem. Specifically, the region leverages implementation research to address key challenges in immunization program complexity. These challenges include lagging immunization coverage across multiple countries, immunization schedules and targets continuing to expand, and policy shifts resulting in dwindling global health funding. Additional challenges are faced across immunization delivery efforts as well, including reliance on “one-size-fits-all” approaches, insufficient use of context specific data, limited innovation and adaptation in response to changing needs, and delayed translation of research into practice. Overall, the region highlighted limitations on how innovative practices are shared across countries make comparisons difficult and limit evidence generation on effectiveness, ultimately limiting success of cross-context replication. The region additionally noted new opportunities to use implementation research in resource-constrained settings, including to:

- Strengthen informed decision-making using implementation evidence
- Enhance country-driven research agendas coordinated at regional level
- Ensure optimal and equitable use of resources for research in prioritization processes
- Enhance local investment and country capacity to conduct research
- Improve cost effectiveness and program efficiency

The region also highlighted two ongoing WHO-led immunization implementation research activities. First, the SP7 Scoping project aims to undertake taxonomy exercise to classify different types of vaccine research, conduct a multi-country landscape analysis assessing decision-making processes using a reference test case, and initiate and leverage a dynamic community of immunization research experts. Second, the MAINSTREAM Initiative aims to support and improve the delivery of Gavi country programs by institutionalizing learning by mainstreaming embedded implementation research in country immunization programs.

While several member states innovating best practices and techniques to improve vaccination over the life course in local settings have begun sharing their experience across peer learning platforms (e.g., Gavi Zero Dose Learning Hub), these innovations are not often shared widely across member states, which make comparisons difficult and limits evidence generation on effectiveness, ultimately limiting success of cross-context replication. As such, the region recognized that there is a need for a common framework, specific to vaccination programs, for documenting and reporting innovative practices from countries during peer learning. As a response, the region developed the Documenting and Reporting Implementation Strategies of Vaccination Efforts (DRIVE) framework for immunization programs. The DRIVE framework has eight main components: naming strategy, problem, context, strategy definition, mechanism of action, implementation strategy specifications and results, facilitators and barriers of successful implementation, additional information.

Finally, challenges with institutionalizing and operationalizing implementation research in immunization were highlighted including insufficient human resources, inadequate funding, and inadequate technical resources.

*Session 2: IA2030 impact modeling*

IVIR-AC has previously reviewed multiple updates from the Immunization Agenda 2030 (IA2030) modeling team to produce estimates for the IA2030 Impact Goal indicator 1.1 (“Number of future deaths averted through vaccination”), which has been used for advocacy and global monitoring and evaluation. The first round of target estimates, produced in 2021, included 14 pathogens and routine immunization data to estimate mortality impact of vaccination (i.e., deaths averted)^1^. The EPI@50 modeling analysis^2^ leveraged the IA2030 framework, but used modified methods with additional model inputs, predictors (e.g., vaccination coverage, stunting, maternal mortality, health spending), temporal extrapolation, multiple functional forms, and estimated vaccine impact by calendar year (instead of year-of-vaccination). Now, at the midpoint of IA2030, the project team is consolidating methods from the original IA2030 estimation framework and the EPI@50 analysis while updating target estimates to include new pathogens, updated model runs from the Vaccine Impact Modeling Consortium (VIMC), mortality and morbidity impact, routine and non-routine immunization data, and updated methods.

The project team provided updates on modeling methods based on previous IVIR-AC recommendations from February 2025, including implementing year-of-vaccination impact calculation ​for select pathogens and reducing the number of potential statistical models and included SDI as a predictor​. Additionally, the project team explored the importance of region and income level and determined that these factors are best captured via mixed effects models (i.e., with income level and WHO region included as random effects) to improve model performance. Particularly for hepatitis B, addition of geographic and economic context via random effects accounted for a higher proportion of observed variation in impact compared to models without these random effects.

The project team has updated the framework to include updated WUENIC vaccination coverage estimates and 2023 VIMC burden estimates, and noted that updates to VIMC models lead to changes in impact factors for VIMC pathogens in the IA2030 midterm estimates.

The project team has also incorporated additional pathogens in the evaluation framework. First, influenza burden (as estimated by the London School of Hygiene and Tropical Medicine^3^) across current and no vaccination scenarios for years 2025 and beyond have been added. These estimates require temporal extrapolation for 2020-2024, and proposed methods included assuming calculating impact ratios for previous years based on vaccination coverage assumptions and cohort size. Polio estimates from KidRisk, includes estimates of paralytic cases, deaths, and DALYs in vaccination and no vaccination scenarios, were included in the evaluation framework. Estimates include doses of both oral and inactivated polio vaccine, but need to be extrapolated beyond year 2023. Proposed extrapolation methods include a similar impact ratio approach proposed for influenza. New VIMC pathogens have been added including malaria, cholera, typhoid, and COVID-19. Preliminary estimates of impact of COVID-19 vaccination have a very high magnitude, likely attributable in part to the applied year-of-vaccination impact calculation.

*Session 3: Immunization coverage estimates – subnational estimates for priority countries*

To identify areas for more targeted support and to monitor subnational progress, there is increased demand for independent WHO/UNICEF estimates of national immunization coverage (WUENIC)-like subnational vaccination coverage estimates in high impact countries (i.e., Democratic Republic of the Congo, Ethiopia, India, Nigeria and Pakistan), ideally to be released annually along with annual WUENIC estimates. The WUENIC team presented “WUESNIC”, which is a WUENIC-like exercise where WHO and UNICEF reviews available data and provides subnational coverage estimates at the first-administrative unit level.

The WUESNIC approach was piloted in an exercise for the Democratic Republic of Congo to estimate coverage at first-administrative unit level​ from 2019-2024 for multiple vaccines (i.e., first- and third-dose coverage of diphtheria-tetanus-pertussis vaccine [DTP1, DTP3], third-dose of pneumococcal conjugate vaccine [PCV3], first-dose of inactivated polio vaccine [IPV1], and first-dose of measles-containing vaccine [MCV1]). Estimates are currently undergoing country consultation, and were generated based on available data, which included a vaccination coverage survey, the 2023 DHS Key Indicator report, and official country estimates. These official country estimates (also referred to as ESNIC) produced via collaborative workshop with representation from EPI, provinces, in-country partners and statistics bureau. Overall, the WUESNIC estimates more closely follow survey data compared to the ESNIC estimates. The pilot exercise highlighted data quality issues across both administrative and survey data, including stock-outs, high drop out rates, underestimated denominators, variability in survey details available, and card availability.

Additional challenges raised during the pilot exercise include subnational data availability and quality across countries (including other countries without official country subnational estimates), WUENIC rules need adapting (e.g., sample size ≥ 300, Grade of Confidence approach, average of WUESNIC may differ from WUENIC), no established process for country consultation and outside of WUENIC cycle, official first-administrative coverage data not requested in the current electronic Joint Reporting Form (eJRF), and a heavy workload in a context of reduced resources. The team shared proposed plans of next steps including supporting countries to perform annual country-level ESNICs, producing WUESNIC estimates for at least 3 priority countries in next 6 months, and continue engagement with the IVIR-AC subgroup on immunization coverage estimation.

To monitoring late or catch-up vaccination (e.g., from the Big Catch-up), WUENIC also is considering to expand the current approach of estimating coverage among the eligible cohort (e.g., surviving infants) to include a cohort-based approach. A challenge to conceptualizing this approach is that there is no globally accepted definition of late, delayed or catch-up vaccination beyond recognition that it though should incorporate some information on the target age of vaccination.

To produce cohort estimates of coverage, the WUENIC team examined data availability by country and region. Overall, high variability in availability was observed across administrative (e.g., occasionally includes multiple age groups) and survey data sources (e.g., includes dated vaccination cards, but only among 12- to 36-month-olds). The team presented proposed next steps including revising the eJRF, finishing analysis of electronic information systems, document issues seen in Big Catch-up countries, and continuing engagement with the IVIR-AC subgroup on immunization coverage estimation.

*Session 4: Modeling diphtheria-tetanus-and-pertussis burden and vaccination strategies*

The DTP Boost tool project team presented the first interactive, live-running web-based decision-making tool that integrates health and economic effects of diphtheria-tetanus-pertussis containing vaccine (DTPCV) booster dose introduction and routine delivery. The team developed the tool to be used in collaboration between modelers and in-country partners to explore scenarios of DTPCV primary series, booster dose and/or maternal vaccination across different schedules, coverage levels and delivery locations.

The tool includes default input data sets (i.e., cases reported in eJRF, WUENIC coverage estimates, United National World Population Projection population sizes, estimated burden from the Global Burden of Disease study); however, tool users can modify data inputs to alternatively include other or local data sources. The team is additionally exploring how to best incorporate seroprevalence data if available. Model calibration is currently within the online tool interface, though could alternatively be conducted offline. Modeled results include outputs of cases, deaths, resource usage (e.g., hospitalizations, outpatient visits), cost-effectiveness and budget impact. Three semi-independent age-structured transmission models are run for each diphtheria, tetanus and pertussis; models are linked via vaccination and population characteristics. Vaccination assumptions include dose dependent coverage, effectiveness and waning. The diphtheria model includes features to allow for both symptomatic and asymptomatic transmission as well as the effect of antibiotic treatment on reductions in colonization. The tetanus model captures environmental transmission and immunity is provided only via multiple vaccine doses. Finally, the pertussis transmission model includes four types of clinical outcome severities and different protection levels by vaccine product (i.e., acellular versus whole cell vaccines).

The project team piloted the DTP Boost tool in Uganda with collaboration from Uganda EPI (UNEPI) and UNITAG. The project team met for multiple in-country workshops and also held virtual meetings. Scenarios explored included how many booster doses to include and the timing of introduction rollout across booster doses (e.g., simultaneous, phased).

From the pilot exercise, the project team highlighted that technical assistance was vital to optimal use of the tool. Additional key considerations emphasized by the project team for ongoing dissemination beyond pilot study include balancing tradeoffs between flexibility and transparency versus ease of use, tailoring to the needs of specific countries, balancing demo tool for functional exploration and engagement versus full tool use, capacity building, connectivity and computing capacity, and handling technical updates.

*Session 5: WHO guidance to translate modelling to support evidence-informed decision-making for immunization*

The IVIR-AC subgroup writing team presented an overview of the newly developed guidance document. The document contains four main chapters. The first chapter (“What are models and how can they help us?”) outlines what are mathematical models, how they can help decision-makers, who uses mathematical models, and how they are integrated in the immunization decision-making process (i.e., Evidence to Recommendation process). The second chapter (“How can policy makers work together with modelers?”) provides readers with an overview of the roles and responsibilities of various members of modeling project teams and best practices for collaboration throughout modeling projects (i.e., communication and respect in the engagement). The third chapter (“How are models made”?) contains a technical overview of the stages of a modeling project, including defining a modeling question, data contextualization, and the modeling process (i.e., choosing model structure, parameter estimation, visualizing uncertainty, model validation, and scenario analysis) and the related key considerations. The fourth and final chapter (“How should modeled results be interpreted and evaluated?”) describes how to evaluate models, including methods, data quality and relevance, uncertainty, interpreting outcomes and scenarios, and the translation process itself (e.g., feedback loops with modelers and model users). Throughout the document, an example of a country interested in introducing HPV vaccination in their immunization schedule is maintained to provide an overarching case study across all aspects of the modeling, evaluation and decision-making process.

The guidance document was reviewed throughout 2025 by regional and national immunization technical advisory group (RITAG, NITAG) members, global and regional WHO experts, and other stakeholders (e.g., academics). Of the 62 reviewers invited, 26 provided detailed feedback that was incorporated into the guidance document presented during the session. Themes of previous reviewer feedback included expanding the glossary, including additional examples, and prioritizing content for the main guidance document versus supplementary material to improved flow and readability.

Key additional considerations presented by the writing team include how best to structure the document from perspective of the decision-maker (e.g., how best to separate model development from evaluation and to separate model evaluation regarding the model itself versus its results), and how best to balance providing gold standard guidance versus guidance tailored to local realities (e.g., co-creation of models with modelers and decision-makers versus decision-makers appraising existing modeling studies). Before the end of 2025, the writing team and IVIR-AC subgroup plan to incorporate recommendations from IVIR-AC and finalize the guidance document. Opportunities for wider impact could also include the development of online web appendix material, accompanying training toolkit (i.e., curriculum built on principles outlined in guidance document), and identifying opportunities to deliver training via NITAG and RITAG engagement.

*Session 6: WHO Multi-Model Comparisons for Typhoid Conjugate Vaccine Adequate Schedules (MMC-TAS)*

A member of the modeling team presented an overview of the four models used for the multi-model comparison (Burnet, IDM, Stanford, Yale). While two models are agent-based and two are compartmental, all include states for susceptible, infected, recovered, acute versus subclinical infection, chronic carriers and vaccination. Each model was run for 12 scenarios, to include the following:

- Two scenarios of waning (i.e., fast-waning and slow-waning)
- Three burden archetypes (i.e., medium, high, and very high incidence)
- Two epidemiologic settings (i.e., Africa, with higher case fatality rates [CFR] and treatment costs, and Asia, with lower CFR and treatment costs)

For each scenario, the following delivery strategies were explored:

- No vaccination,
- Single dose routine vaccination (i.e., 9 [or 15] months, 2 years, 5 years),
- Single dose routine vaccination and boosters (i.e., 9 [or 15] months & 5-year-old booster only, 9 [or 15] months & 5- and 10-year-old boosters)

Overall, model comparisons showed that the Burnet model predicted the lowest burden averted, the IDM and Yale models predicted (similar) higher burden averted, and the Stanford model predictions varied across settings. The main driver identified to explain differences between models was the average duration of vaccine-induced immunity (i.e., IDM and Yale models used parameters from statistical model fit to vaccine efficacy data, while Burnet and Stanford models re-calibrated parameters in transmission model as part of validation, with Burnet estimating a considerably shorter duration of immunity for both the fast- and slow-waning scenarios).

Over a 10-year time horizon, per 100,000 people in a high incidence setting with approximately 200 cases per 100,000 people per year, TCV introduction is predicted to avert 250-1,500 cases (5-45 cases per 1,000 doses), ​15-300 hospitalizations, 0.5-32 deaths​, and 25-2,000 DALYs​. In medium incidence settings (i.e., 50 cases per 100,000 person-years), routine vaccination at 2 or 5 years (with a catch-up campaign) may be cost-effective in Africa; TCV introduction is not likely to be cost-effective in Asia. Meanwhile, in high incidence settings (i.e., 200 cases per 100,000 person-years), TCV introduction is very likely to be cost-effective; delaying routine introduction until 2-years-old or adding a booster is the optimal strategy if waning is fast in Africa; in Asia, TCV introduction may be cost-effective but the optimal strategy is to delay vaccination until 2- or 5-years-old. Finally, in very high incidence settings (i.e., 1,500 cases per 100,000 person-years), TCV introduction is cost-saving and adding a booster dose is very likely to be cost-effective in Africa; in Asia, TCV introduction is cost-saving but the optimal strategy is to delay vaccination until 2 years of age or add a booster dose.

At a willingness-to-pay threshold of $2,500 (i.e., the average gross domestic product [GDP] per capita for a lower-middle-income country), TCV introduction is cost-effective when incidence is greater than 10-60 cases/100,000 person-years in Africa and greater than 75-200 cases per 100.000 person-years in Asia. Delaying routine vaccination schedules (until 2- or 5-years-old) is more cost-effective at lower willingness to pay thresholds and lower incidence levels. Routine vaccination at 9-months-old and a booster dose at 5-years-old is cost-effective when typhoid incidence is greater than 100-250 cases per 100,000 person-years in Africa ​ and greater than 500-1,000 cases per 100,000 person-years in Asia​.

*Session 7: Novel metrics to value combination vaccines*

The project team presented three key areas of development to assess novel metrics to value combination vaccines: a non-systematic literature review, consultations with stakeholders, and a convening of health economic experts.

The non-systematic literature review was conducted with a goal to establish a baseline understanding of how combination vaccines have been evaluated to date. Findings from the review identified standard health economic metrics that were used across studies, including reductions in cases, vaccination program costs, cost per disability-adjusted life year (DALY) averted, and cost per Quality-adjusted life year (QALY) gained. The review identified that evaluations were conducted to compute the standard incremental benefits and costs of each additional antigen rather than the comprehensive value added by the combination. Also, evaluations would only account for the impact of all components of a vaccine when the combination under consideration is focused on adding a strain or valent (e.g., HPV, influenza). However, identified sources often mentioned other benefits of combination vaccines without directly evaluating them.

Consultations with stakeholders were conducted to develop a list of benefits, value drivers and potential metrics that can be used to more fully capture the value of combination vaccines. A key theme from the consultations was that it is necessary to account for risks of combination vaccines, not just benefits. Additionally, many stakeholders commented on the importance of cost, market fragmentation and the loss of autonomy over their immunization schedule (if standalone vaccines were to become unavailable). Following the consultations, additional benefits and risks were added to the list under consideration by project team.

Finally, the project team convened a group of health economic experts to prioritize value drivers and metrics based on four criteria (resonance, magnitude, quantifiability, and fit within existing frameworks). They held a two-day virtual meeting of 15 experts from academia and international organizations, including WHO and UNICEF, in June 2025. Key output from the meeting included a prioritized “checklist” of consensus benefits, value drivers, and metrics for consideration when evaluating combination vaccines. Feedback from the meeting focused on the classification change for various value drivers and metrics, suggestions to add clear definitions for criteria, revisions of value driver list to avoid redundancy, adjustments to language, and additions to the list (e.g., metrics for an equity value driver). Each value driver was assessed for resonance and magnitude and each metric by quantifiability and fit within existing frameworks in a three-color stop-light system (i.e., red, yellow, green).

The ultimate goal of the project is to apply prioritized value drivers and metrics to future combination vaccines via PATH and partner modeling of select metrics for prioritized combination vaccines. Next steps identified by the project team included developing a manuscript describing the results of the literature review, approach to develop and prioritize value drivers and metrics list and checklist​, and also applying these metrics in future analyses.

*Session 8: Stockpiling and vaccination strategies for Ebola response: insights from integrated epidemiological and epidemic modeling*

The modeling team presented results from updates to an individual-based transmission model. Alongside a costing model, the transmission model was used to simulate health outcomes under different counterfactual scenarios to explore the benefits, costs, and cost-effectiveness of different interventions for Ebola outbreaks, including vaccination. Contact between individuals in the model were based on households, extended family networks, hospitals, and others in the community. Interventions explored included healthcare worker vaccination (including both preventative and reactive) and others related to case finding, including contact tracing, vaccination (including both ring or geographical strategies), Ebola Treatment Unit admissions, and safe and dignified burials.

This model is not calibrated, and is instead run using stochastic simulations with some parameters fixed and some varied. The model was parameterized using demography and geography of the Democratic Republic of the Congo and using information on Ebola natural history and costs from the literature. Parameters explored across ranges of worse-case, realistic and best-case include transmission (R_0_), case ascertainment timeliness and coverage, contact tracing performance, and vaccination coverage.

The model was used to explore five main questions. Findings for each question are as follows:

- *Is the Ervebo vaccine cost-effective against Ebola virus disease?*

Model results suggest that reactive ring vaccination tends to be cost-saving if at least one large outbreak occurs during a 5-year period.

- *What is the most beneficial vaccination ​strategy – ring or geographic?​*

The modelled results show that improving standard response activities is more critical than vaccination strategy​; however, on average a geographic strategy uses fewer doses in worst-case response scenarios and that a ring strategy uses fewer doses in realistic and best-case response scenarios.

- *Should we preventively vaccinate ​healthcare workers?​*

Modeled results suggest that the cost-effectiveness of vaccinating healthcare workers preventatively depends mostly on the frequency of outbreak emergence. Additionally, waning immunity and healthcare worker turnover also affect the cost-effectiveness of this strategy.

- ​*How many doses should be maintained in ​the stockpile?​*

Model results suggest that the optimal stockpile size depends on the level of accepted risk and the speed of dose replenishment. ​

- *Is the current price of an Ervebo ​dose justified?​*

Across model simulations, the vaccine is either very valuable component of the outbreak response, or not a very useful addition to existing measures​. The percentage of five-year projection horizons for which the value-based price per Ervebo dose is greater than $98​ ranges between 50% to 70% over different willingness-to-pay thresholds. Overall, modeled results suggest that if the aim is to be prepared for a large EVD outbreak occurring within the next 5 years, the current price of an Ervebo dose is justified.​

*Session 9: Malaria multi-model comparison of prioritized interventions (M3CPI)*

A member of a modeling team presented plans for methods for the multi-model comparison. Two models (i.e., malariasimulation and OpenMalaria) will be used to simulate of interventions across sampled locations. Setting-specific input characteristics will be included in the model, such as infection history, intervention efficacy, and geographic characteristics. The overall approach is based on the concept that economic analyses, model integration and archetype definitions may lead to policy-relevant generalizable patterns to inform global WHO guidance.

Data inputs to the modeling process include seasonality of malaria transmission, vector composition and behavior population age distribution, history of interventions deployed, and history of malaria burden. To reproduce historical interventions and burden, simulations will be run for each first-administrative unit level setting (n=600) from 43 countries of sub-Saharan Africa. Each subnational setting reflects intervention and malaria transmission history and provides a common basis for model alignment and comparison across scenarios. Future scenarios analyzed include business as usual, scaling up or interruption of interventions. Interventions, to be used in combination, include insecticide treated nets (ITNs), indoor residual spraying (IRS), chemoprevention (i.e., seasonal malaria chemoprevention [SMC] or perennial malaria chemoprevention [PMC]), malaria case management, and vaccination. Across each setting, 24 combination scenarios were selected for simulation and outputs (e.g., malaria cases, deaths and DALYs averted) will be computed over short- and long-term time horizons. Outputs will be combined with estimated costs of intervention for cost-effectiveness analysis. The cost database was generated via a literature review that was conducted by WHO; the literature review identified 95 studies before 2022 and additional 60 studies from SR up to 2025.

Proposed visuals for how results will be presented across scenarios include ranked Sankey diagrams by probability of cost-effectiveness, with x-axis of scaled down to up of interventions. Moving forward, results will be presented and finalized in November 2025 during the project’s 3rd Technical Consultation.

*Session 10: Additional seasonal doses of malaria vaccine (7 doses vs. 5 doses)*

The project team at PATH presented a model from Imperial College London that was most recently reviewed by IVIR-AC in 2024. This model has previously demonstrated the positive health impact of malaria vaccine in seasonal settings across 3, 4 and 5 dose schedules. Vaccine efficacy was estimated using a model parameterized to account for uncertainty observed in matching trial outcomes during model validation to the extended RTS,S and seasonal malaria chemoprevention (SMC) Phase 3b trial data (where children had received 7 doses of vaccine, with or without SMC). The main transmission model parameterized over range of transmission levels and a 15-year time horizon across two seasonal settings (i.e., highly seasonal and seasonal) and different monthly cycles of SMC (i.e., 4 and 5 cycles). The model assumes constant treatment coverage and other vector control interventions across the projection period. Vaccination coverage scenarios include different vaccine campaigns and dropout rates.

Vaccine delivery scenarios include seasonal and hybrid strategies for both primary (i.e., doses 1 through 3) and additional doses (i.e., doses 4 through 7).

The preliminary modeled results suggest that across a range of seasonal and transmission settings, both 5-dose and 7-dose RTS,S schedules show positive public health impact and cost-effectiveness relative to no vaccination. The addition of doses 6 and 7 increases the cumulative number of cases and DALYs averted. Additionally, cost-effectiveness (ICERs) remained similar between 5- and 7-dose schedules, as additional costs of extra doses offset extra cases averted.​

An analysis of different dose allocation strategies was conducted to compare the impact and cost effectiveness of vaccinating fewer children with 6 or 7 doses versus vaccinating more children with 5 doses. Preliminary results suggested that when the doses were used to reach more children with a 5-dose schedule, there are generally improvements in health outcomes in children under 5. However, the models suggest that the allocation of additional doses for use in a 5-dose schedule does not provide significant additional benefits when measured in children up to age 10.​

*Session 11: 3 doses vs. 4 doses of malaria vaccine in perennial settings*

The project team from Imperial College London presented a malaria transmission model fit across transmission settings that accounted for age patterns of prevalence and disease outcomes. The RTS,S model was originally fit to 4-year follow-up data from the Phase 3 trial, involving age-based vaccination in six low-transmission and five high-transmission sites. The R21 model was fit to 3-year follow-up data from the Phase 2b trial in a study with highly seasonal transmission. This calibrated model was validated against data on vaccine efficacy against clinical malaria in the R21 Phase 3 trial, with 2 year of follow-up data available to date. The R21 Phase 3 trial included data from two sites with highly seasonal transmission where vaccination was administered before the malaria season, and three sites receiving age-based vaccination (two sites with low/moderate perennial transmission and one site with moderate and seasonal transmission).

The project team from The Kids presented the OpenMalaria model, which is an individual-based stochastic model calibrated against age patterns of disease and parasite density. The Kids RTS,S model was fit to RTS,S Phase 3 trial data, using age-disaggregated case counts in 11 trial sites including both perennial and seasonal settings. The R21 model was fit to incidence data from the 30-month follow-up timepoint from the R21 Phase 3 trial, and the calibrated model was validated against the incidence of uncomplicated malaria observed in control and vaccination arms of the Phase 3 trial, as well as the vaccine efficacy against clinical malaria by study site.

Both models were also assessed for alignment with age patterns of severe malaria in the MVIP and the vaccine efficacy against severe malaria from the MVPE case-control analysis (conducted in the MVIP vaccination clusters in Ghana, Kenya and Malawi). These included comparisons of vaccine efficacy of 3 doses versus 0 zero doses at 0.5–6, 6–18, and 18+ months following the third dose; 4 doses versus zero doses at the same time intervals following the fourth dose; and 4 doses versus 3 doses following the fourth dose.

**References**

1. Carter, A. *et al.* Modeling the impact of vaccination for the immunization Agenda 2030: Deaths averted due to vaccination against 14 pathogens in 194 countries from 2021 to 2030. *Vaccine* (2023) doi:10.1016/j.vaccine.2023.07.033.

2. Shattock, A. J. *et al.* Contribution of vaccination to improved survival and health: modelling 50 years of the Expanded Programme on Immunization. *The Lancet* **403**, 2307–2316 (2024).

3. Goodfellow, L. *et al.* The potential global health impact and cost-effectiveness of next-generation influenza vaccines: A modelling analysis. *PLOS Medicine* **22**, e1004655 (2025).
